# Valuation of a Company Producing and Trading Seaweed for Human Consumption: Classical Methods vs. Real Options

**DOI:** 10.3390/ijerph18105262

**Published:** 2021-05-15

**Authors:** Raisa Pérez-Vas, Félix Puime Guillén, Joaquín Enríquez-Díaz

**Affiliations:** 1 Faculty of Economics and Business, IC2-ECOBAS, Universidade de Vigo, Lagoas-Marcosende s/n, 36310 Vigo, Spain; 2 Faculty of Economics and Business, University of A Coruña, 15008 A Coruña, Spain; felix.puime@udc.es (F.P.G.); joaquin.enriquez@udc.es (J.E.-D.)

**Keywords:** discount cash-flow, real options, aquaculture, human consumption

## Abstract

Aquaculture is an increasingly relevant sector in the exploitation of natural resources; therefore, it is appropriate to propose various models that include the fundamental variables for its economic-financial valuation from a business point of view. The objective of this paper is to analyze different models for the valuation of investment projects in a company in the aquaculture sector in order to conclude whether there is a model that represents a better valuation. Therefore, in this study, four valuation models have been applied, three classical models (net present value, internal rate of return, and payback) and a more recent model, real options (RO) for a company producing and marketing seaweed in Galicia (region located in the northwest of Spain). The results obtained, RO (€5,527,144.04) and net present value (€5,479,659.19), conclude that the RO model estimates a higher added value by taking into account in its calculations the flexibility given by the expansion option. Future lines of research include the application of valuation models that have been applied to companies belonging to the same sector in order to compare whether the results found are similar.

## 1. Introduction

Aquaculture is an increasingly important sector worldwide. In recent years, aquaculture production has grown constantly, with an increase of 25.7% compared to 2000 with 2018 [1]. Aquaculture provides the market with the main source of aquatic products [2]. The world’s aquaculture production comes from farms that raise fish, crustaceans, seaweed, mollusks, and other invertebrates [3].

In the European Union, aquaculture represents an important economic activity [2]. Spain is the European Union country with the highest aquaculture harvest with 347,825 tons (25.5% of the total). However, in terms of production value, it ranks fourth with 478.8 million (11% of the total) [3]. In addition, aquaculture production is increasingly being developed by countries to achieve a sustainable aquaculture industry [1]. In this line, the search for new sustainable sources of production makes the production of microalgae become relevant in possible sustainable applications (e.g., under input requirements) [4]. In addition, algae biotechnology generates environmental and economic benefits since it mitigates climate change and helps control environmental pollution [5].

The inclusion of new food products in the human diet is becoming more and more common. The current importance of a healthy diet leads to the inclusion of healthy foods [6]. Edible seaweed provides proteins, amino acids, minerals, and vitamins in addition to possessing therapeutic potential in disease prevention [7,8]. The consumption of seaweed is widespread in Eastern countries, although its consumption is not widespread in Western countries [7,9,10,11], there is a trend towards its increase in the Western diet [12,13]. In Spain, there is no tradition of seaweed consumption; therefore, it is considered a new food product [9,14] that is increasingly present in supermarkets [11]. The incursion that seaweed consumption is gradually having in Spain leads to the importance of assessing the viability of seaweed producing companies.

The relevance that aquaculture is having leads to the importance of the valuation of companies in this sector. Numerous studies carry out the valuation of investment projects in terms of sustainability [15,16,17,18,19,20]. The evaluation of projects in the seaweed industry sector is scarce; therefore, its study is relevant. Company valuation is a fundamental tool in corporate finance. Various valuation models have been studied for decades, the most widely used being models based on discounted cash flows (DCF) such as net present value (NPV) or internal rate of return (IRR). However, these models have certain shortcomings that have been alleviated by the real options (RO) approach. Numerous investigations have contrasted the RO model as a new methodology to overcome the shortcomings of the classical models. The field where the first applications of RO began to be developed is natural resources. Tourinho [21] studies the option of temporary abandonment of the project or definitive abandonment of natural resource reserves. Trigeorgis [22] makes the valuation of canceling the project during construction, abandonment, and expansion of production on the extraction of minerals. Armstrong et al. [23] address the treatment of uncertainty in the development of oil exploitation through the study of the option of obtaining more information, due to the irreversible nature of the decision to invest in oil exploitation. Nelson, Howden, and Hayman [24] perform an application of RO through decision trees on the conservation of natural properties taking into account climate change as uncertainty. Wang and Du [25] perform a development of the binomial method through RO to evaluate investment decisions in carbon capture and storage in coal-fired power plants in China. In the field of R&D is McGrath and Nerkar [26] which expose that RO are a successful methodology to evaluate new options in R&D in the pharmaceutical industry. In the same field, Magazzini, Pammolli, and Riccaboni [27] also investigate on the role of RO in R&D projects in the pharmaceutical industry. Levaggi, Moretto, and Pertile [28] use the RO approach to investigate on the benefits that can be achieved through the development of new pharmaceutical products. Research conducted on the application of the RO methodology on the energy sector are the most numerous in the field of RO, currently focusing on renewable energies, although studies such as the one conducted by Venetsanos, Angelopoulou, and Tsoutsos [29] have already dealt with wind energy production in Greece. Davis and Owens [30] analyze through RO the value of renewable electricity technologies in the face of uncertain fossil fuel prices. Wesseh and Lin [31] discuss RO valuation of wind power technologies in China to assess the feasibility of wind power projects.

More recently, the applications of RO are carried out in different areas: mining [32,33,34,35]; airport [36,37,38]; participation-public-private (PPP) [39,40]; agriculture [41,42,43]; and pharmaceutical [44,45,46,47,48,49]. However, in the aquaculture sector, research in this area is very scarce. The evaluation of projects in the seaweed industry sector is also scarce, therefore, its study is relevant. The objective of this paper is to value through classical valuation models (NPV, IRR, and Pay-Back) and the more current RO approach a seaweed trading company based in Galicia (Spain) to analyze if they lead to similar results or not. The rise of the RO methodology in other sectors highlights the great interest of this approach; therefore, the validity of this approach in the aquaculture sector will be contrasted. RO is suitable for sectors with high uncertainty [50,51,52]. RO considers management flexibility. The value of this flexibility plays an essential role in the face of the uncertainty of the project environment [53].

The article is structured as follows: Section 2 contains the most important valuation methods. Section 3 explains a description of the case study. Section 4 provide the results of the application of the different models. Section 5 addresses the discussion. Finally, Section 6 presents the conclusions.

## 2. Materials and Methods

There is a variety of models for the valuation of investment projects. The most widely used models are those based on discounted cash flows (DCF) such as NPV or IRR [54]. In addition, another traditional model is the payback. More recently, and due to the rigidity of the classic models that do not take flexibility into account, a new model has emerged to value investment projects, RO.

### 2.1. Net Present Value

NPV is a classic valuation model that bases its calculation on the cash flows of an investment project. The purpose of this method is to find out whether or not it is feasible to carry out a project. This method is accepted for projects whose future income is guaranteed and is known with certainty before the valuation [36]. The calculation of NPV is relatively simple since it depends on four variables [55]. The discount rate of the cash flows is the weighted average cost of capital (WACC). Mathematically

(1) $$NPV=-C+\overset{T}{\underset{t=1}{\sum }}\frac{R_{t}}{{\left(1+r\right)}^{t}}$$

where:

*C* = initial investment

$R_{t\;}$= cash flows

*r* = discount rate (WACC)

*t* = number of periods

The basic rule of thumb for this method is to carry out the project if NPV > 0. Only those investments whose capital value is positive should be carried out, since they are the only ones that contribute to the achievement of the company’s general objective, in other words, to increase the value of the company. When there are several investments with a positive capital value, priority should be given to those with the highest capital value. This criterion provides a measure of the expected return in absolute (€) and present value.

This model has the advantage of considering the different maturities of cash flows. The disadvantage is the complexity of estimating the WACC, since this is a weighting of the cost of debt and equity, and it is precisely in the estimation of the cost of equity where the difficulty may lie, since there may be certain volatility in the value of the shares.

### 2.2. Internal Rate of Return

The return on investments can also be measured through the IRR, which relates the return on the asset to the cost of the corresponding liability. IRR is used to measure the profitability of a project in relative terms. This model calculates the internal rate of return that makes the NPV equal to 0 [54].

(2) $$0=-C+\overset{T}{\underset{t=1}{\sum }}\frac{R_{t}}{{\left(1+IRR\right)}^{t}}$$

This model provides the minimum rate of return for a project. Through this model it is concluded that a project is profitable when

IRR>r (WACC)

### 2.3. Payback

The payback is an indicator of how long it takes to recover the investment [56]. In more detail, it is the time it takes for the initial investment (A) to be recovered (amortized).

When net cash flows (*FC*) are constant, *FC1 = FC2 = FCn = FC*, the payback period is defined by the formula

(3) $$Payback=\frac{A}{FC}$$

When net cash flows are not constant, *FC1 ≠ FC2 ≠ FCn ≠ FC*:

$$A=\overset{P}{\underset{j=1}{\sum }}FC_{j}$$

According to this model, the most interesting project is the one with the shortest payback period.

Among the disadvantages of this model are: (1) it does not consider the net cash flows obtained after the recovery period; (2) it does not consider the difference in the maturities of the net cash flows obtained before reaching the payback period; and (3) it therefore prioritizes or rewards those projects that involve a faster disinvestment, inspired by a policy of liquidity rather than profitability.

### 2.4. RO

The RO approach captures an implicit value in the investment project that the NPV is unable to capture [57]. RO captures that value through options. The term “RO” has its origin with Myers [58]. This methodology is based on the concept of option finance theory. An option is the right to buy or sell an asset in a specified period. This theory extrapolates this term to the real assets of the company. RO can include decisions to make, to abandon, to expand or to contract a capital investment within or at a specific time [51]. These options are known as managerial flexibility and increase the value of projects [59]. Through this methodology, project managers must see uncertainties as opportunities to create value [60]. The classical NPV, IRR, and payback models present limitations to capture the value of the uncertainty [61]. There are various valuation models, B-S, Binomial decision tree model. Due to the clarity of the binomial decision tree model, it is the one that will be used to carry out the valuation of the investment project.

The RO methodology is closely linked to NPV. RO is an extension of NPV; therefore, the new value is called Expanded NPV or Strategic NPV [50,62,63,64,65,66,67]

(4) STRATEGIC NPV=NPV+OPTION VALUE

The option values provide information to decision makers to select whether the option maximizes the value of the project or, if there is more than one option, which one provides more value. The value of the option is calculated through the binomial decision tree model, a discrete time model proposed by Cox et al. (1979) [68]. This model has been adapted to make binomial trees that collect the value of the options.

Considering the underlying asset *S*, *σ* the volatility of the underlying asset, the duration of the option *T*, the number of periods of the binomial tree n, *dt = T/n*, the upward movement of the underlying asset u and the downward movement of the underlying asset *d*, for the calculation of each node of the binomial tree of the underlying asset

(5) $$u=e^{\sigma \sqrt{dt}}$$

(6) $$d=e^{-\sigma \sqrt{dt}}=\frac{1}{u}$$

(7) $$\sigma =ln\left(\frac{\sum_{i=1}^{n}S_{i}}{\sum_{i=0}^{n}S_{i}}\right)$$

The value of the underlying asset for node *i, j* can be generalized as

(8) $$S_{i,j}=u^{i}d^{\left|i-j\right|}S_{0,0}$$

In addition, for the creation of the binomial tree corresponding to the option, it is necessary to calculate the probabilities, $p_{u}$ probability neutral to the risk of an increase in the value of the underlying asset, and $p_{d}$ probability neutral to the risk of a decrease in the value of the underlying asset

(9) $$p_{u}=\frac{e^{r_{f}\frac{T}{n}}-d}{u-d}$$

(10) $$p_{d}=1-p_{u}$$

where $r_{f}$ is risk free rate.

The value of the option is defined by

(11) $$C=\left(p_{u}C_{u}+p_{d}C_{d}\right){\left(1+r_{f}\right)}^{-dt}$$

where $C_{u}$ and $C_{d}$ represent the value of the option if the underlying asset increases or decreases, respectively.

## 3. Project Description

### 3.1. Case Study: Application to a Seaweed Production Company

The cultivation of seaweed is increasingly widespread as shown in Table 1; therefore, companies need valuation models that help them make better decisions and adapt to new events.

In Spain, according to data from the Ministry of Agriculture, Fisheries and Food [69], tons of seaweed are produced for marketing. Table 1 shows the latest production data and its value [70].

The choice of an aquaculture company for this study has been motivated by: first, the boom that this sector has been having in recent years; second, as they are relatively new companies, they need greater flexibility as soon as new information that affects them appears, therefore, it is important to apply models such as the RO that provide flexibility; third, future lines of research will seek extrapolation to similar sectors (for example, aquaculture); fourth, in other fields where natural resources are dealt with, RO research is widespread, which is why, a priori, it is considered adequate for this sector.

2018 was considered as the base year, with the total assets for that year being €5,479,659.19, this value being equivalent to the investment carried out by the company as of 31 December 2018. Subsequently, the income statement, balance sheets and cash-flows generated by the company for the 2019–2023 years were estimated. To calculate the cash-flows, the operating profit/loss was used. These were deducted the tax on profits, to later add depreciation and changes in working capital. This cash-flow obtained is adequate for the valuation of the company. As these cash-flows are at different times in time, it is necessary to have an update rate, in our case, the WACC.

Considering that it is not intended to liquidate the company in 2023, a continuation value was calculated through the Gordon–Shapiro model

(12) $$Continuation\;value=\frac{Last\;cash-flow}{WACC-g}$$

where *g* is the growth rate

The calculation of the WACC was based on the financial structure of the company, the cost of external financing and the return demanded by the shareholders.

Table 2 shows how cash flows are generated from the financial statements and projected financial information.

### 3.2. RO Model

Through the three previous model’s flexibility is not being considered. This flexibility is incorporated through the expansion option. The evaluation of the project is carried out through several stages: first, valuation of the project by means of the construction of the binomial tree relative to NPV; second, evaluation of the project through the construction of the binomial tree considering the flexibility (expansion option); third, valuation of the option.

For the valuation using RO, the variables described in Table 3 are necessary.

The parameters necessary for the use of RO are defined:

(1) Discount rate: has been used the weighted average cost of capital (WACC)
(2) Risk-free rate $\left(r_{f}\right)$: is the risk-free interest rate, for the study the interest rate of a 10-year Spanish Bond dated 23 February 2021 has been taken.
(3) Net Present Value (NPV): in the RO methodology it represents the underlying asset.
(4) Volatility *σ*: measures the variability of cash flows. For its calculation, the logarith-mic present value returns [71] has been used.
  (13) $$\sigma =ln\left(\frac{\sum_{i=1}^{n}CF_{i}}{\sum_{i=0}^{n}CF_{i}}\right)$$
(5) *u*: represents the rise factor of the underlying asset.
(6) *d*: represents the downside factor of the underlying asset.
(7) $p_{u}$: risk neutral probability of upward movement.
(8) $p_{d}:$ probability neutral to the risk of downward movement.
(9) Exercise price: represents the option strike price. In this case it represents the investment carried out in the investment project. Asset depreciation estimates have been used to estimate it.
(10) Expansion factor: indicates the growth factor of the company.
(11) Time step: is the period of each stage. As an annual cash-flow valuation is carried out, the variation between the variable is one.

## 4. Results

This section presents the results of the case study, including the valuation using the three classical models and the valuation using RO, including the construction of the binomial trees.

### 4.1. Valuation Models Classics

The valuation of the project was carried out using two models based on cash flows, NPV and IRR, and also using payback.

Table 4 shows the results obtained in the evaluation of the project through the classical models. This project estimated by the NPV shows a value greater than 0 which indicates benefits in the company. The good results reflected above and the growth expectations since it is a booming sector, cause a valuation of €5,479,659.19. This fact is also observed in the calculation of the IRR, providing a profitability of 20.42% being well above the value of the WACC, which shows the high profitability of this type of companies. The latest classic studio model, payback provides 11-year payback.

### 4.2. RO Results

The growth potential of the company has been studied by evaluating the incursion of an expansion option. This flexibility value has not been taken into account in the valuation models described above.

To calculate the value of the project using RO, two binomial trees have been built: the binomial tree relative to NPV shown in Table 5 and the binomial tree, considering the expansion option shown in Table 6 For its construction, the parameters described in Table 3 have been necessary.

The first step has been the creation of the binomial tree relative to the underlying asset (NPV) shown in Table 5. Its construction starts from the initial node relative to period 0, whose value is the NPV previously calculated. Subsequent nodes are calculated by upward movements u and downward movements d. The analysis shows a great volatility in the value of the project in the period of completion (period 5) due to the great difference of values obtained in the last nodes, being a maximum value of €140,778,311.39 in the case of growth in all periods and of €213,290.42 in the worst case, that is, when there is a drop in value in all years.

The binomial tree relative to the value of the project with the expansion option shown in Table 6 begins to be built by the final nodes through a process called backward induction. These nodes are calculated using a maximization rule of NPV vs. the value of the project give consideration the flexibility. Therefore, the project value at the node, relative to $NPV_{5,1}$ is defined by $max\left(NPV_{0}*u^{5};NPV_{0}*u^{5}*F-P\right)$. The rest of the end nodes are calculated using the same equation.

For the calculation of intermediate nodes, the maximization rule of carrying out the expansion in this node or updating the value of the next previously updated node is applied.

(14) $$RO_{i,j}=Max\left(NPV_{i,j}F-P;\;p_{u}NPV_{i+1;j+1}+p_{d}NPV_{i,j+1}\right){\left(1+r_{f}\right)}^{-dt}$$

As in Table 5, the binomial tree relating to the value of the project taking into account the expansion option shows a great disparity in the value of the nodes of period 5. This circumstance is since the construction of this tree depends on the values obtained in the binomial tree relating to the NPV. The value of the expansion option is defined by

(15) Value expansion option=5,527,144.04−5,479,659.19=47,484.85€

The value of the incursion of flexibility in the investment project lies in €47,484.85, therefore, having the company the decision-making capacity to expand in each period, where the information is more up-to-date, provides a positive value to the business.

## 5. Discussion

Considering the importance for humanity to reduce its impact on the planet in terms of emissions and pollution, so that in today’s civilization we can establish a sustainable model in which man can develop his life cycle without harming the planet, the generation of food in an environmentally friendly way is vital.

If we consider that the planet is mostly covered by seawater, whose natural wealth is unquestionable, we must find the answer to the problem of sustainable human food from aquaculture, and more specifically from edible seaweed. However, seaweeds are not included as a staple food in the diet of most of the world’s peoples and cultures. This is of course largely due to the general lack of knowledge about the nutritional properties of seaweed and its contribution to human health. On the other hand, in market economies, where investors make decisions to participate and develop an economic sector in terms of profitability, seaweed farming as a business activity also sounds quite far removed from the more usual investment decisions. Therefore, shedding light on the viability of this type of activity, using appropriate investment valuation models, will give more confidence to potential investors by eliminating much of the initial uncertainties that may hover over the investors’ minds.

For this purpose, a classical valuation of an investment project to produce seaweed for human consumption was initially carried out, using the classical payback, IRR, and NPV models. Although these models yielded very positive values in terms of investment feasibility, it was decided to perform a new valuation under the RO approach to capture an implicit value in the investment project that classical models such as NPV are not able to observe.

In fact, the RO model gives the uncertainties that exist in every investment project, motivated by variables that are difficult to measure, an inherent value and an opportunity to create value that classical models do not contemplate. A great deal of research has been carried out in this field. Santos et al. [72] conclude that the valuation performed by RO through binomial trees is superior to that obtained by NPV in estimating the valuation of a hydroelectric plant. In this line, the study by Zhang, Zhou and Zhou [65] concludes that RO analysis is more effective than NPV under conditions where uncertainty exists. The RO methodology is suitable for valuing an Internet company in China [73]. Adetunji and Owolabi [74] expound that the application of the RO approach in railway infrastructure is better than discounted flow-based models as it incorporates the value of flexibility. Ajak and Topal [60] conclude that flexibility incorporating RO approach through binomial decision trees into the valuation increases the value of the project. Polat and Battal [36] expose that the results of valuing a project in view of the value of an option is more optimistic than valuation using traditional models. The results obtained in this research show that the RO approach through binomial trees also offers a higher value than the NPV valuation, although the values obtained are very similar, which confirms the validity of the data obtained in the first valuations.

This will undoubtedly give more consistency to the fact that the cultivation of seaweed for human consumption is not only a sustainable solution for feeding mankind, but also a viable and very profitable economic activity.

There are certain limitations in our research. The valuation of investment projects is based on estimates of a multitude of interrelated parameters. One of the limitations of the study is that the cash flows necessary to carry out the valuation are projections that may be affected in the future by changes that may occur in the socio-economic, environmental, and technological fields. Although the real options approach has tried to minimize the effects of a possible alternative scenario, there is always a non-controllable part. Another limitation lies in the calculation of the rate of capital cost (WACC) since it is being calculated for a limited period, although the parameters used in its estimation vary throughout the life of the project; this limitation opens the possibility of a new line of research. Finally, the scarcity of articles on the valuation of aquaculture companies through RO does not allow for a comparative analysis that would be feasible in other sectors where this approach is more common.

## 6. Conclusions

Over the last few decades of the 20th century, Galicia experienced a great development of the aquaculture sector [75]. Initially as a complementary branch of the already consolidated fishing industry, but due to the biophysical and hydrodynamic characteristics of the region, as well as the established institutional framework, it has increased its relative importance in the production of maritime-fishery products for human consumption [76]. In addition, diversity and high innovation and development in aquaculture nowadays have led to the cultivation of other species. This is the case of seaweed farming for human consumption. In Spain, this sector has tripled its production in the 2016–2019 period, and its growth is expected to reach even higher quotas, turning this sector into an industry of the future [69].

As any industry, investment is necessary for its development. The agents who usually make decisions on whether or not to carry out investments have to make decisions in uncertain environments seeking a minimum profitability. Accordingly, this article presents the valuation and profitability of a seaweed production company in Galicia (Spain). This valuation has been carried out using three classical valuation models—NPV, IRR, and payback—and a new approach, RO. The analysis carried out in this article shows that the results obtained by the four proposed models lead to a main conclusion about the profitability of the company studied: there is no doubt about its positive profitability.

The RO approach is a methodology increasingly applied by academia whose case studies focus on sectors with a high level of uncertainty. The application of this model in comparison to classical valuation models in the aquaculture sector, although it has been addressed by academia, is a field of study that has not yet reached maturity. Therefore, in this study, several valuation models have been applied to verify if there is a discrepancy or not between the different methodologies.

The results show that the valuation through the RO (€5,527,144.04) provides a slightly higher result than that obtained with the VAN (€5,479,659.19) due to the inclusion of flexibility measured via an expansion option. Despite the addition of this option and the slightly higher valuation, both models provide very similar values. Furthermore, a conclusion is drawn across all four valuation models: it is profitable to invest. Therefore, it is assumed that the valuation obtained is reliable and this will undoubtedly give a boost to potential investors to invigorate the seaweed aquaculture sector. Furthermore, the contribution made in this analysis will help decision-makers to consider the RO approach as a valid and reliable methodology for the appraisal of investment projects. Moreover, the results of the model presented here provide the foundation on which legislative proposals can be formulated to encourage the co-responsibility of stakeholders in decision-making.

For this reason, it is necessary to establish new research approaches to develop a theoretical framework that addresses the needs of the economic and social agents involved in business activity. As mentioned in the introduction, RO have been applied to sectors such as energy, pharmaceuticals, mining, agriculture, and public–private partnerships. These industries have been characterized by significant uncertainty due to the high degree of volatility of future results. However, the great possibilities offered by the RO methodology turn it into a transdisciplinary approach that can be applied to emerging industries such as, for example, startups and all those sectors with a great innovation component associated with a high degree of uncertainty. Among them is the aquaculture sector. Aquaculture is distinguished by the diversity of its crops, production methods, and geographical location. The multiplicity of these characteristics is not an obstacle for the RO to be applied to different aquaculture case studies. This would determine which productions would be the most profitable within the aquaculture sector and stimulate its growth by reducing the degree of uncertainty faced by potential investors.

## Figures and Tables

**Table 1:** Value of seaweed production in Spain.

Year	Value (€)	Tons of Seaweed Production
2016	1,049,500	6.83
2017	1,468,312.92	7.32
2018	2,120,734.18	11.129
2019	2,004,445	18.586

Source: Ministry of Agriculture, Fisheries and Food [50].

**Table 2:** Cash flow projection for a five-year time horizon (€).

	2019	2020	2021	2022	2023
Revenue	4,760,021.00	4,760,021	4,998,022.05	5,247,923.15	5,510,319.31
Cost of goods sold	−3,282,323	−3,094,013.65	−3,248,714.33	−3,411,150.05	−3,581,707.55
Personnel expenses	−507,584	−507,584	−532,963.2	−559,611.36	−587,591.93
Other expenses	−691,623	−691,623	−726,204.15	−762,514.36	−800,640.08
Depreciation expenses	−147,871	−147,871	−147,871	−147,871	−147,871
Operating profit/loss	130,620	318,929.35	342,269.37	366,776.39	392,508.76
Operating profit/loss × (1 − T)	116,251.8	239,197.01	256,702.03	275,082.29	294,381.57
Depreciation expenses	147,871	147,871	147,871	147,871	147,871
Stocks variation	196,476	245,966.82	−32,229.31	−33,840.77	−35,532.81
Customer variation	166,685	16,370.72	−43,687.86	−45,872.26	−48,165.87
Cash-flow	483,532.8	478,430.79	351,966.91	367,716.87	384,254.33
Cash-flow Gordon–Shapiro	10,278,750.28				

**Table 3:** RO variables.

Parameter	Value
(1) Discount rate	3.74%
(2) Risk-free rate $r_{f}$	0.36%
(3) Net Present Value (NPV)	€5,479,659.19
(4) Volatility σ	64.92%
(5) u	1.91
(6) d	0.52
(7) $p_{u}$	0.3457
(8) $p_{d}$	0.6543
(9) Exercise price P	€1,478,710
(10) Expansion Factor F	1.05
(11) Time step	1

**Table 4:** Valuation of the seaweed company using classical models.

Valuation Model	Value
Net present value	€5,479,659.19
Interest rate of return	20.42%
Payback	11 periods

**Table 5:** Net Present Value binomial tree (€).

0	1	2	3	4	5
					140,778,311.39
				73,549,429.94	
			38,425,795.78		38,425,795.78
		20,075,502.73		20,075,502.73	
	10,488,418.05		10,488,418.05		10,488,418.05
5,479,659.19		5,479,659.19		5,479,659.19	
	2,862,840.21		2,862,840.21		2,862,840.21
		1,495,686.82		1,495,686.82	
			781,419.47		781,419.47
				408,251.50	
					213,290.42

**Table 6:** Real Option binomial tree (€).

0	1	2	3	4	5
					146,338,516.96
				75,753,994.93	
			39,285,032.18		38,868,375.57
		20,405,984.67		20,228,107.75	
	10,614,208.64		10,541,083.61		10,488,418.05
5,527,144.04		5,497,862.81		5,479,694.62	
	2,869,149.04		2,862,877.22		2,862,840.21
		1,495,715.83		1,495,696.49	
			781,429.57		781,419.47
				408,254.14	
					213,290.42

## Data Availability

Data is contained within the article.
